# Severe Pulmonary Bleeding after Assist Device Implantation: Incidence, Risk Factors and Prognostic Impact

**DOI:** 10.3390/jcm11071908

**Published:** 2022-03-29

**Authors:** Bernd Panholzer, Kevin Pilarczyk, Katharina Huenges, Charlotte Aldinger, Christine Friedrich, Ulrike Nowak-Göttl, Jochen Cremer, Assad Haneya

**Affiliations:** 1 Department of Cardiovascular Surgery, University of Schleswig-Holstein, 24105 Kiel, Germany; bernd.panholzer@uksh.de (B.P.); katharina.huenges@uksh.de (K.H.); charlotte.aldinger@uksh.de (C.A.); christiane.friedrich@uksh.de (C.F.); jochen.cremer@uskh.de (J.C.); assad.haneya@uksh.de (A.H.); 2 Department of Intensive Care Medicine, Imland Klinik Rendsburg, 24768 Rendsburg, Germany; 3 Institute of Clinical Chemistry, University of Schleswig-Holstein, 24105 Kiel, Germany; ulrike.nowak-goettl@uksh.de

**Keywords:** LVAD, heart failure, bleeding, hemoptysis, pulmonary hemorrhage

## Abstract

Background: Continuous flow left ventricular assist devices (CF-LVAD) improve survival in patients with advanced heart failure but confer risk of bleeding complications. Whereas pathophysiology and risk factors for many bleeding complications are well investigated, the literature lacks reports about pulmonary bleeding. Therefore, it was the aim of the present study to assess incidence, risk factors, and clinical relevance of pulmonary bleeding episodes after LVAD implantation. Methods: We retrospectively analyzed our institutional database of 125 consecutive patients who underwent LVAD implantation between 2008 and 2017. Demographic and clinical variables related to bleeding were collected. The primary endpoint was incidence of severe pulmonary bleeding (SPB). Results: Nine out of 125 patients suffered from SPB during the postoperative course (7.2%) 11 days after surgery in the median. None of them had a known history of lung disease or bleeding disorder. History of prior myocardial infarction (0% vWD. 42.2%, *p* = 0.012) and ischemic cardiomyopathy (25.0% vs. 50.0%, *p* = 0.046) were less frequent in the SBP group. Concomitant aortic valve replacement was more common in the group with SPB (33.3% versus 7.0%, *p* = 0.034). Surgical (blood loss 9950 vs. 3800 mL, *p* = 0.012) as well as ear-nose-throat (ENT) bleedings (33% vs. 4.6%, *p* = 0.015) were observed more frequently in patients with SPB. SPB was associated with a complicated postoperative course with a higher incidence of acute kidney failure (100% versus 36.7%, *p* = 0.001) and delirium (44.4% versus 14.8%, *p* = 0.045); a higher need for red blood cell (26 packs versus 7, *p* < 0.001), fresh frozen plasma (18 units versus 6, *p* = 0.002), and platelet transfusion (8 pools versus 1, *p* = 0.001); longer ventilation time (1206 versus 171 h, *p* = 0.001); longer ICU-stay (58 versus 13 days, *p* = 0.002); and higher hospital mortality (66.7% vs. 29%, *p* = 0.029). Conclusion: SPB is a rare but serious complication after LVAD implantation and is significantly associated with higher morbidity and mortality. The pathophysiology and potential risk factors are unknown but may include coagulation disorders and frequent suctioning or empiric bronchoscopy causing airway irritation.

## 1. Introduction

Due to the increasing shortage of appropriate donor organs with an expanding number of patients on the waiting list for a heart transplantation; an improvement in applicability, durability, and patient selection and management, the use of long-term mechanical circulatory support devices, especially left ventricular assist devices (LVADs), has become an accepted treatment option for patients with end-stage heart failure [1]. Third-generation continuous flow left ventricular assist devices (LVADs) have recently been introduced in clinical routines [2].

However, non-surgical major complications can frequently be observed after LVAD implantation in the early postoperative period as well as long after discharge. These major adverse events mainly include bleeding complications, device thrombosis, ischemic and hemorrhagic strokes, acute kidney injury, multi-organ failure, and infections, all of them associated with a significant increase in morbidity and mortality [3]. With an incidence ranging between 20% and 60%, bleeding is the most common complication in LVAD patients and can occur at different sites including surgical bleeding, GI bleeding, or intracranial bleeding [4]. Pathophysiology of hemorrhagic complications is multifactorial and not fully understood, but includes device hemocompatibility, development of GI angiodysplasia, and vascular malformations due to non-pulsatile flow and acquired coagulopathies such as von Willebrand disease (VWD) or platelet dysfunction as well as antiplatelet and anticoagulant therapy. This may also be owing to the presence of thrombocytopenia and comorbidity, including renal failure, further impairing platelet function, and previous need for ECMO support pre-LVAD implantation [5,6]. However, pathophysiology varies between different bleeding types and sites, thus, identifying the potential causes and risk factors for bleeding is essential to be able to prevent and treat bleeding complications sufficiently and to improve outcome after LVAD implantation.

Whereas mild pulmonary bleeding seems to be a common condition in patients undergoing general cardiac surgery with extracorporeal circulation as well as in patients with systemic anticoagulation, there are almost no reports in the current literature describing incidence and prognostic impact of early postoperative severe pulmonary bleeding in patients undergoing LVAD implantation for advanced heart failure. Therefore, we performed this retrospective analysis aiming to analyze incidence, risk factors, and morbidity and mortality associated with postoperative pulmonary hemorrhage.

## 2. Materials and Methods

### 2.1. Study Population

In this retrospective investigation, we analyzed 125 consecutive patients who underwent LVAD implantation between April 2008 and September 2017. Among this cohort, we identified nine patients (7.2%) who developed severe pulmonary bleeding (SPB) postoperatively. SPB was defined as intrapulmonary hemorrhage, confirmed by bronchoscopy, which did not suspend spontaneously and required blood transfusion.

### 2.2. Inclusion and Exclusion Criteria

Patients with end-stage cardiomyopathy aged 18 to 99 years undergoing LVAD implantation at our department of cardiac surgery were included into the study.

### 2.3. Study Aim

The aim of the study was to review relevant pre-, intra-, and postoperative variables such as preexisting conditions, operative parameters, vasopressor and inotropic support, hemostasis and anticoagulation regimens, postoperative morbidity (duration of intensive care unit (ICU) stay, ventilation time, renal function, etc.), and hospital mortality to uncover predictors and outcome relevance of SPB.

### 2.4. Statistics

Continuous pre-, intra-, and postoperative variables and patient characteristics are presented as median and 25th and 75th percentiles and for age with additional mean and standard deviation. The continuous data of patients with SPB and no SPB were compared by Mann–Whitney U-test. Categorical variables are summarized as absolute (n) and relative (%) frequencies and compared by Fisher’s exact test. Missing data were excluded pairwise and stated in the tables when exceeding 5%. Survival of the patient groups during FU was estimated on right-censored data by Kaplan–Meier analysis, presented as survival-curves, and survival of the SPB and no-SPB subgroups was compared by log-rank test.

All tests were conducted two-tailed and a *p*-value of ≤5% was considered statistically significant. Data were analyzed with IMB SPSS statistics for Windows (Version 26.0).

### 2.5. Ethics

The present study was approved by the Institutional Ethic Review Board (EK D 496/18) and informed consent from the patient or the patient’s next of kin was obtained.

### 2.6. Surgical Procedure

All operations were carried out under general anesthesia by experienced surgeons as described previously [7]. In the majority of cases, the surgery was performed via median sternotomy; in individual cases, minimally invasive surgery was performed via left-sided minithoracotomy and superior ministernotomy. Cardiopulmonary bypass was necessary in all cases, but especially within recent years and in the absence of concomitant procedures, the operation has been performed beating heart and with induced ventricular fibrillation instead of obtaining myocardial protection with a cold blood cardioplegic solution. Continuous CO_2_ insufflation was used to avoid cardiac air embolism. LVAD implantation was performed with common techniques: after suturing the fixation ring on the apex of the left ventricle, the entry into the left ventricle was made by coring the myocardium with circular knife. Thereafter, the driveline was externalized in the left or right upper quadrant of the abdomen, and the outflow graft was anastomosed end-to-side to the ascending aorta. LVAD was started at a low rate of revelations and a thorough de-airing was performed; residual air in the left ventricle was ruled out by transesophageal echocardiography.

## 3. Results

Among the 125 included patients, we identified 9 patients (7.2%) whose postoperative course was complicated by SPB. The median onset time of SPB was 11 (2; 15) days after surgery, and duration of SPB was 11 (7; 33) days.

### 3.1. Comparison of Patients with SPB vs. Patients without SPB

A comparison of preoperative findings and demographic variables between patients with and without SPB is presented in Table 1. Whereas age, gender, comorbidities, and other demographics did not differ between study groups, incidence of prior myocardial infarction (0% in patients with SPB vs. 42.2% in patients without SPB, *p* = 0.012) and ischemic cardiomyopathy as underlying diseases (25.0% vs. 50%, *p* = 0.046) was lower in patients with SPB.

A comparison of intraoperative data between patients with and without SPB is presented in Table 2. Whereas urgency of surgery, length of surgery, cardiopulmonary bypass, and aortic cross clamping as well as other variables of surgical procedure and perioperative management did not differ between groups, frequency of aortic valve replacement as concomitant procedure was higher in patients with SPB (33.3% in patients with SPB vs. 7% in patients without SPB, *p* = 0.034).

**Figure 1:**
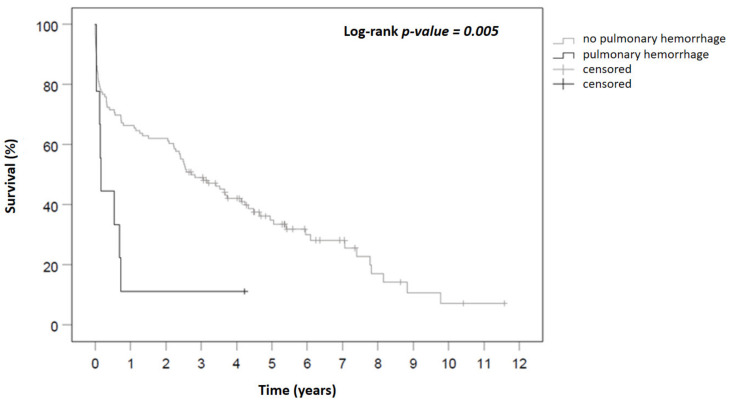
Kaplan–Meier Survival Curve.

A comparison of postoperative data and outcomes between patients with and without SPB is presented in Table 3. Within the first 24 h, the demand for transfusion of blood products did not differ significantly, except for platelets being administered more often in patients in SBP. The overall demand for blood and coagulation products (PRBC: 26 (23; 46) in patients with SPB vs. seven (3; 17) in patients without SPB, *p* < 0.001; FFP: 18 (12; 32) in patients with SPB vs. six (3; 13) in patients without SPB *p* = 0.002, platelets: eight (2.5; 12.5) in patients with SPB vs. one (0; 3) in patients without SPB, *p* = 0.001) throughout the hospital stay was significantly higher in the group of the patients who developed SPB. Accordingly, surgical blood loss via drainages was higher in patients with SPB. In line to a very complicated postoperative course, a significant higher incidence of acute kidney injury (AKI) according to KDIGO classification (100% in patients with SPB vs. 36.7% in patients without SPB, *p* < 0.001), and a significantly higher need for renal replacement therapy (RRT, 100% in patients with SPB vs. 24.7% in patients without SPB, *p* < 0.001) could be observed in the SPB group. Patients with SPB suffered from a postoperative delirium (44% in patients with SPB vs. 14.8% in patients without SPB, *p* = 0.045) as well as bleeding in the ENT region (33% in patients with SPB vs. 4.6% in patients without SPB, *p* = 0.015) more frequently. Length of stay in the ICU was significantly longer in the group of patients who suffered from SPB (58 (29.5; 71) days in patients with SPB vs. 13 (6; 33) days in patients without SPB, *p* = 0.002). Ventilation time was also longer among this cohort (1206 (810; 1330) hours in patients with SPB vs. 171 (22; 783) days in patients without SPB, *p* = 0.001). 30-day mortality did not differ between the groups, contrary to in-hospital mortality, which was more than twice as high in the SPB group (66.7% in patients with SPB vs. 29% in patients without SPB, *p* = 0.029) (Figure 1).

A comparison of laboratory values is presented in Table 4. Besides hemoglobin levels, measured directly after admission to ICU, we found no significant differences.

### 3.2. Management of SPB and Anti-Coagulation after SPB

According to our institutional protocol, initial management of SPB included temporary stopping of aspirin and heparin or warfarin were stopped. After cessation of SPB, heparin was restarted at a low dose and slowly increased, targeting lower activated partial thromboplastin time (APTT) levels until a safe cessation of SPB was achieved. A description of blood and coagulation products administered to the patients is presented in Table 5. Due to secondary anemia, median transfusion of 19 units of packed red blood cells (PRBC) was necessary during the period of SPB. To increase hemostasis, median transfusion of 13 units of fresh frozen plasmas (FFP) and 6 units of platelets was necessary; after further examinations of coagulation ability were conducted, median substitution of 2400 units of prothrombin complex concentrate (PCC), 4 grams of fibrinogen and 6000 units of coagulation factor XIII were applied. For further clarification of an acquired von Willebrand syndrome (AVWS) an analyses of VWF:Ac/VWF:Ag ratio was made, showing us that AVWS was present in all patients, and median application of 19,000 units of factor VIII/von Willebrand factor concentrates was performed.

## 4. Discussion

To the best of our knowledge, there is only one study including 25 patients undergoing LVAD-implantation available in the literature reporting about severe pulmonary bleeding events in patients after LVAD implantation. Whereas incidence, pathophysiology, risk factors, and management of other bleeding complications, e.g., GI or cerebral bleeding, after LVAD implantation are well investigated, data about pulmonary bleeding complications even after general cardiac surgery or during mechanical circulatory support are rare [5,6].

Aubron et al. reported on overall incidence of bleeding events of 60% and an incidence of 10% of pulmonary bleeding in computed tomography scan and bronchoscopy under veno-venous or venoarterial ECMO support for lung or heart failure. The study could show a significant association of the severity of illness assessed by the APACHE III score as well as high aPTT levels on the day before bleeding occurs [8].

Welp et al. performed a retrospective study including 25 patients who underwent LVAD implantation and additional temporary RVAD support due to perioperative right heart failure. The need for right ventricular support for more than 7 days and a blood flow of more than 4 L/min were both associated with pulmonary bleeding complications [9]. All bleeding events occurred more than 7 days after surgery. 

In a retrospective single-center cohort study from a Dutch university hospital, pulmonary bleeding occurred in 8% of patients during treatment with extracorporeal membrane oxygenation. Pulmonary bleeding occurred on day 7 (2−11). Independent risk factors for the occurrence of hemorrhagic complications were a higher mean aPTT in the 24 h prior to the bleeding event, duration of mechanical lung or circulatory support, and mode of mechanical support (venoarterial ECMO = circulatory support) [10].

Pathophysiology and risk factors contributing to bleeding after LVAD implantation vary significantly between the types of bleeding. Bleeding that occurs during the early phase after surgery may be mainly related to the surgical intervention as postsurgical bleeding. Thus, the surgical trauma itself, preoperative characteristics of the patients, e.g., bleeding disorders, and the initiation of anticoagulant and antiplatelet therapy are involved in the pathogenesis of bleeding [11]. Bleedings that occur late after surgery, mainly after the first 14 days, are non-surgical and are mainly located in the GI tract and the central nervous system. Underlying mechanisms are complex and diverse: device hemocompatibility, the need for aggressive anticoagulation to prevent thrombosis, development of GI angiodysplasia and vascular malformations as a consequence of non-pulsatile LVAD flow, or acquired coagulopathies induced by the mechanical stress of the LVAD, e.g., as von Willebrand disease (VWD) or platelet dysfunction [12,13]. In addition, postoperative systemic infections can lead to coagulation activation and disseminated intravascular coagulation. Many patients with advanced heart failure suffer from thrombocytopenia and acute kidney injury further impairing coagulation [12,13]. 

Based on the rare published data about pulmonary bleeding in LVAD-patients and our study, pathophysiological mechanisms leading to or triggering pulmonary hemorrhage may be multifactorial and remain mainly unclear. A variety of conditions can cause hemoptysis in the general population, including RV failure, coagulopathy (anticoagulant-related), thrombocytopenia, liver dysfunction, disseminated intravascular coagulation, bronchitis, airway trauma, a foreign body, and infection [14]. As the PTT was associated with the bleeding event in two studies, anticoagulation may play a role in the development of pulmonary bleeding. Anticoagulant-induced pulmonary bleeding in the general population is a rare clinical entity and has been infrequently documented in the medical literature. A recently published review revealed 18 studies documenting 22 patients, that suffered from hemoptysis under therapy with warfarin, DOACS or antiplatelet therapy [15]. The additional use of antiplatelet therapy to oral anticoagulation had an important contribution to this complication. As the anticoagulation regime after LVAD implantation always consists of a very aggressive anticoagulative therapy including heparin and antiplatelet therapy, this may be at least one of the trigger factors for endobronchial bleeding events. However, in our study, coagulation labs were not associated with bleeding events. Whereas blood loss in the first 24 h—as an indicator of surgical bleeding—was comparable between both groups, the higher blood loss via surgical drainages in total in patients with SPB as well as higher incidence of bleeding in the ENT region may suggest that anticoagulation or systemic coagulation disorders prone the patients to bleeding from different locations including the surgical site as well as the lung. One major coagulation disorder involved in bleeding complications in LVAD patients is the acquired vWD. As this condition cannot be assessed with routine lab works, it can be hypothesized that—in accordance with other bleeding complications in LVAD-patients—it is at least partly involved in the pathogenesis of SPB.

Recently published studies support the evidence that hemodynamic changes under LVAD support alter systemic angiogenic signaling and cause abnormal angiogenesis in mucosal tissues [16]. Consecutively, LVAD support can induce nasopharyngeal hypervascularization that might be possibly linked to vascular changes that can be observed during gastrointestinal angiodysplasia in LVAD patients, leading to GI bleeding. The detailed pathophysiologic mechanisms of these changes in the nasopharyngeal area have not been described in the literature. In addition, it is unclear if this phenomenon can also be observed in lung tissue. As the pulmonary bleeding events occurred early, with a median of 11 days after surgery, it appears at least to be unlikely that formation of AV-angiodysplasia plays a crucial role in the development of pulmonary bleeding complications.

The higher prevalence of concomitant replacement of the aortic valve in patients with SPB might be an observation without causative link. As bioprostheses were used and due to the need for anticoagulation in all LVAD patients, aortic valve replacement does not add any anticoagulative effect in general. Combined procedures prolong length of surgery and CPB and thus might affect coagulation. However, in our series, CBP time was 40 minutes longer in the SBP groups without being statistically significant. Indication for aortic replacement was regurgitation in all cases. Severe aortic regurgitation can lead to post-capillary pulmonary hypertension due to raised left ventricular end-diastolic pressure increasing the risk for pulmonary bleeding.

All of our patients were monitored perioperatively with a pulmonary artery catheterization (PAC). The incidence of pulmonary artery rupture during PAC is relatively low, ranging from 0.01–0.47% in the literature [17]. Despite its low incidence, associated mortality is considerably high, being over 70%. Mechanical trauma of the vessel wall with consecutive rupture can be caused by the catheter tip being directed into the vessel wall or be a result of catheter migration to a smaller caliber branch with subsequent rupturing during balloon inflation. The mechanism of injury can be explained by the use of stiff catheter tips, the repeat manipulations of PA catheter during cardiopulmonary bypass, and the excessive inflation of the balloon. In most cases, there is one major injury with massive bleeding, whereas our bronchoscopy findings showed multiple small lesions with diffuse hemorrhage, making the PAC appear unlikely to play a causal role in our patients.

Pulmonary hypertension is reported to be the underlying cause of hemoptysis in 0.2–4% of cases [18]. Hemoptysis may be a result of pulmonary infarction or rupture of dilated pulmonary arteries, arterioles, or aorticopulmonary collateral vessels. Although bleeding events are usually mild, more severe events can occur from pulmonary vascular disease with pulmonary hemorrhage or rupture of aneurysmal pulmonary arteries. Hemostatic mechanisms that contribute to bleeding include thrombocytopenia, abnormal platelet function, clotting factor deficiencies, and depletion of von Willebrand factor. However, pulmonary bleeding complications do not translate to a relevant increase in mortality in patients with PAH, with sudden cardiac death and/or right-sided heart failure being the most common causes of death in these patients. As PH can frequently be observed in LAVD patients, this comorbidity might at least be involved in the pathogenesis of pulmonary bleeding and its high incidence in this cohort. However, incidence of PH as well as severe RV failure with need for temporary RV support was comparable between study groups in our cohort. The diagnosis of PH was mainly based on the noninvasive estimation of pulmonary artery systolic pressure in patients with tricuspid regurgitation by doppler echocardiography as we did not perform routine preoperative right heart catheterization. Therefore, we cannot reliably differentiate between pre- and post-capillary PH. It can be at least hypothesized that the majority of patients suffered from post-capillary PH due to left-sided heart failure, but we cannot prove this hypothesis. In our cohort, incidence of AKI was higher in patients with SPB. It cannot be stated that AKI is a consequence of SBP, if SBP is a consequence of AKI, or if AKI is just a bystander and a surrogate marker of very compromised and ill LVAD patients with a higher generalized bleeding risk.

Patients with perioperative bleeding after cardiac surgery are characterized by an increased risk of AKI [19]. Bleeding with hemodynamic instability and need for re-exploration are independent predictors of AKI. In addition, anemia with a low arterial oxygen content can lead to low renal oxygen delivery. Blood transfusion can cause a direct transfusion-related kidney injury. On the other hand, the need for transfusion of blood products might only act as a surrogate marker for hypotension or decreased oxygen delivery [20]. Research demonstrated that patients with acute kidney injury can also suffer from coagulation system disorders due to uremia or anticoagulation during renal replacement therapy. Taken together, it remains unclear if AKI caused an increased bleeding risk or if bleeding, with all its clinical consequences, induced AKI in our study.

Prolonged ventilation with tracheostomy was more frequent in patients with SPB. It cannot be clearly stated if long ventilation is a consequence or the cause of pulmonary bleeding. As most patients without SPB were already extubated before SBP occurred, it seems more reasonable to suggest that long-term ventilation with need for regular suctioning and bronchoscopy might have induced small lesions in a vulnerable lung due to comorbidities and coagulation disorders. Endotracheal suction is a risk factor for pulmonary bleeding, other investigations showed an incidence of hemorrhagic secretion of more than 30% [21]. In our institution, endotracheal suction is commonly performed by closed suction systems, which do not require any disconnection of the ventilation system. This leads to a reduced loss in lung volume compared to open systems. Due to this benefit, usage of closed suction is recommended for patients with high FiO_2_, high PEEP, or acute respiratory distress syndrome. Furthermore, closed systems allow suction without the risk for potential release of infectious—or at least germ-laden—air in a very simple and time-saving procedure. Despite all these advantages, it can be hypothesized that usage of closed systems as a simple and time-saving method that requires no special hygienic measures might increase the frequency and possibly facilitate the occurrence of pulmonary bleeding. Deppe et al. observed a significant increase in number of daily suctions in the closed tracheal suction group and suggested that this was due to the ease of the procedure [22].

Maggiore et al. showed that frequent suctioning of more than six times per day leads to increased risk of bleeding [23]. In addition, we assume that deep suction is performed more often, which, compared to shallow suction, causes tissue trauma of the tracheal or bronchial mucosa and leads to more adverse events.

Some studies about GI bleeding in LVAD patients reported an association of LVAD type and incidence of bleeding events, but the results are inconsistent [24,25]. During the observation period, we used the HVAD^®^ (HeartwareVAD, Medtronic, Dublin, Ireland) Heartmate^®^ II and Heartmate^®^ 3 (Abott, Chicago, IL, USA) in our center with the majority of patients receiving the HVAD (more than 80%). All patients that suffered from SPB were patients placed on the HVAD^®^. However, due to the small portion of patients receiving other systems and the low incidence of SPB, the difference observed did not reach statistical significance and it cannot be proved if there is any link between LVAD system type and risk of pulmonary bleeding.

## 5. Conclusions

In summary, SPB is a rare complication after LVAD implantation and is associated with morbidity and mortality. The causes are multifactorial and heterogeneous, indispensable, and unavoidable, and coagulation deficits lead to massively increased bleeding tendencies, possibly facilitated by alterations in bronchial mucosa. Mechanical stress or damage, caused by endotracheal suction or bronchoscopy, might trigger bleeding in vulnerable tissue.

## 6. Limitations

This is a retrospective, single-center study with design-specific limitations. Due to the relatively small sample size, further studies are necessary to confirm our findings. 

## Figures and Tables

**Table 1:** Preoperative findings and demographic variables.

	Total (n = 125)	No SPB (n = 116)	SPB (n = 9)	*p*-Value
Age, years	61 (52; 67)	61 (52; 66)	65 (50; 73)	0.194
Female gender	20 (16.0%)	17 (14.7%)	3 (33.3%)	0.156
Logistic EuroSCORE (%)	44.1 (32.0; 60.0)	42.5 (32.0; 59.9)	49.6 (39.5; 75.4)	0.221
Additive EuroSCORE (%)	14 (12; 16)	14 (12; 16)	15 (12.5; 16)	0.456
Body mass index, kg/m^2^, (%)	25.4 (22.6; 29.5)	25.3 (22.6; 29.9)	25.4 (22.6; 27.5)	0.771
INTERMACS level	3.0 (2.0; 4.0)	3.0 (2.0; 4.0)	3.0 (2.0; 3.0)	0.060
Acute cardiac decompensation	77 (65.3%)	73 (66.4%)	4 (50%)	0.446
Prior cardiac decompensation	109 (94.8%)	102 (96.2%)	7 (77.8%)	0.070
Arterial hypertension	68 (55.3%)	62 (54.4%)	6 (66.7%)	0.730
Pulmonary hypertension	65 (57.0%)	62 (58.5%)	3 (37.5%)	0.286
**Heart rhythm**				
Sinus rhythm	66 (57.9%)	63 (60.0%)	3 (33.3%)	0.163
Atrial fibrillation	29 (25.4%)	25 (23.8%)	4 (44.4%)	0.229
Other	19 (16.8%)	17 (16.3%)	2 (22.2%)	0.646
Diabetes mellitus	37 (29.6%)	35 (30.2%)	2 (22.2%)	1.000
Hyperlipoproteinemia	59 (50.4%)	56 (51.9%)	3 (33.3%)	0.322
Chronic renal insufficiency	76 (61.3%)	69 (60.0%)	7 (77.8%)	0.480
Decompensated renal insufficiency	22 (17.9%)	20 (17.5%)	2 (22.2%)	0.662
Chronic dialysis	7 (5.6%)	7 (6.1%)	0 (0%)	1.000
COPD	14 (11.3%)	14 (12.2%)	0 (0%)	0.596
Currently smoking	18 (15.1%)	17 (15.5%)	1 (11.1%)	1.000
Previous smoking	38 (32.8%)	37 (34.6%)	1 (11.1%)	0.268
**Coronary heart disease (CHD)**	64 (54.2%)	61 (56.0%)	3 (33.3%)	0.298
No CHD	54 (45.8%)	48 (44.0%)	6 (66.7%)	-----
One-vessel disease	15 (12.7%)	15 (13.8%)	0 (0%)	-----
Two-vessel disease	12 (10.2%)	12 (11.0%)	0 (0%)	-----
Three-vessel disease	37 (31.4%)	34 (31.2%)	3 (33.3%)	-----
Prior myocardial infarction	49 (39.2%)	49 (42.2%)	0 (0%)	0.012
Previous PCI (+/-DES)	49 (39.2%)	46 (39.7%)	3 (33.3%)	1.000
Previous thoracic surgery	44 (35.8%)	40 (35.1%)	4 (44.4%)	0.720
Peripheral vascular disease	14 (11.4%)	14 (12.3%)	0 (0%)	0.596
Clinical presentation				
Acute myocardial infarction (<14 d)	5 (4.2%)	5 (4.6%)	0 (0%)	1.000
Cardiogenic shock (<14 d)	14 (11.9%)	12 (11.0%)	2 (22.2%)	0.289
CPR (<24 h)	7 (5.9%)	6 (5.5%)	1 (11.1%)	0.435
Transfer from intensive care unit	59 (50.0%)	53 (48.6%)	6 (66.7%)	0.490
Intubated prior surgery	25 (21.2%)	23 (21.1%)	2 (22.2%)	1.000
**Cardiomyopathy**				0.046
ICM	58 (48.3%)	56 (50.0%)	2 (25.0%)	-----
DCM	56 (46.7%)	52 (46.4%)	4 (50.0%)	-----
HCM	1 (0.8%)	1 (0.9%)	0 (0.0%)	-----
Others	5 (4.2%)	3 (2.7%)	2 (25.0%)	-----
Acute myocarditis	15 (12.1%)	14 (12.2%)	1 (11.1%)	1.000
Coagulation disorder	7 (5.7%)	7 (6.1%)	0 (0%)	1.000
Apoplex preoperative	13 (10.4%)	13 (11.2%)	0 (0%)	0.596
Neurologic disease	1 (0.8%)	1 (0.9%)	0 (0%)	1.000
**LVAD type**	125 (100%)	116 (110%)	9 (100%)	-----
HVAD	102 (81.6%)	93 (80.2%)	9 (100%)	-----
HM2	12 (9.8%)	12 (10.3%)	0 (0%)	-----
HM3	11 (8.9%)	11 (9.5%)	0 (0%)	-----
RVAD (temporary)	6 (4.9%)	6 (5.3%)	0 (0%)	1.000
BiVAD	1 (0.8%)	1 (0.9%)	0 (0%)	1.000

INTERMACS= Interagency Registry for Mechanically Assisted Circulatory Support, HM = Heartmate, BiVAD = biventricular assist device, ICM = ischemic cardiomyopathy, DCM = dilatative cardiomyopathy, HCM = hypertrophic cardiomyopathy, CPR = cardiopulmonary resuscitation, PCI = percutaneous coronary intervention, DES = drug eluting stent, COPD = chronic obstructive pulmonary disease.

**Table 2:** Comparison of Operative Data.

	Total (n = 125)	No SPB (n = 116)	SPB (n = 9)	*p*-Value
**Urgency**				0.506
Elective	89 (82.4%)	83 (83.0%)	6 (75.0%)	-----
Urgent	15 (13.9%)	13 (13.0%)	2 (25.0%)	-----
Emergency	4 (3.7%)	4 (4.0%)	0 (0.0%)	-----
Length of surgery, min	249 (200; 316)	249 (200; 311)	290 (188; 476)	0.435
Cardiopulmonary bypass time, min	118 (98; 158) h	118 (99; 145)	149 (93; 246)	0.492
Cross-clamp time, min	71 (60; 86)	71 (60; 85)	91 (56; 137)	0.287
Circulatory arrest, min	0 (0; 0)	0 (0; 0)	0 (0; 0)	-----
Number of PRBC	2 (1; 5)	2 (1; 5)	2 (1; 9)	0.657
Number of fresh frozen plasma	2 (0; 6)	2 (0; 6)	1 (0; 7)	0.975
Number of platelets	2 (1; 2)	2 (1; 2)	2 (1; 2)	0.618
Fibrinogen	3 (0; 4)	3 (0; 4)	4 (2.5; 7.5)	0.181
PCC	87 (72.5%)	80 (72.1%)	7 (77.8%)	1.000
PCC (IE)	2400 (2000; 4000)	2500 (2000; 4150)	2400 (1800; 2400)	0.065
Coagulation factor XIII	24 (20.0%)	21 (18.9%)	3 (33.3%)	0.381
Coagulation factor XIII (IE)	1875 (1250; 2500)	2500 (1250; 2500)	1250 (1250; 1250)	0.172
Novoseven	4 (3.3%)	4 (3.6%)	0 (0.0%)	1.000
Novoseven (mg)	6.5 (5.3; 15.3)	6.5 (5.3; 15.3)	0 (0; 0)	-----
**Surgical procedure**				
CABG	4 (3.3%)	4 (3.5%)	0 (0%)	1.000
Aortic valve replacement	11 (8.9%)	8 (7.0%)	3 (33.3%)	0.034
Tricuspid valve replacement / repair	3 (2.4%)	2 (1.8%)	1 (11.1%)	0.205
PFO-closure	7 (5.7%)	6 (5.3%)	1 (11.1%)	0.421
Other				
Ventavis	3 (2.5%)	3 (2.7%)	0 (0%)	1.000
NO	52 (43.3%)	47 (42.3%)	5 (55.6%)	0.499
Perfan	40 (33.6%)	37 (33.6%)	3 (33.3%)	1.000
Adrenalin	111 (92.5%)	102 (91.9%)	9 (100%)	1.000
Milrinone	113 (94.2%)	104 (93.7%)	9 (100%)	1.000
ECLS	25 (20.8%)	23 (20.5%)	2 (25.0%)	0.671

PRBC= packed red blood cells, PCC = prothrombin complex concentrate, ECLS = extracorporeal life support, NO = nitric oxide, CABG = coronary artery bypass grafting.

**Table 3:** Comparison of Postoperative Data and Outcomes.

	Total (n = 125)	No SPB (n = 116)	SPB (n = 9)	*p*-Value
Drainage loss, <24 h postoperative (mL)	850 (600; 1213)	850 (600; 1175)	930 (675; 1800)	0.307
Drainage loss, total (mL)	4100 (2100; 9220)	3800 (2063; 8575)	9950 (6325; 16770)	0.012
Number of packed red blood cells, <24 h	2 (0; 3)	2 (0; 3)	3 (1.5; 5)	0.053
Number of fresh frozen plasma, <24 h	4 (0; 7)	4 (0; 6)	7 (2.5; 15)	0.051
Number of platelets, <24 h	0 (0; 1)	0 (0; 1)	1(0; 2)	0.041
Number of packed red blood cells, total	8 (4; 20)	7 (3; 17)	26 (23; 46)	<0.001
Number of fresh frozen plasma, total	6 (3.3; 14.8)	6 (3; 13)	18 (12; 32)	0.002
Number of platelets, total	1 (0; 5)	1 (0; 3)	8 (2.; 5; 12.5)	0.001
Noradrenalin at admission ICU (µg/min)	0.23 (0.07; 0.62)	0.21 (0.07; 0.61)	0.42 (0.23; 0.85)	0.123
Noradrenalin 1. POD (µg/min)	3.9 (0; 20)	2.00 (0.00; 20.00)	22.0 (5.0; 48.0)	0.099
Adrenalin at admission ICU (µg/kg/min)	0.05 (0.02; 0.09)	0.04 (0.02; 0.09)	0.08 (0.02; 0.10)	0.365
Adrenalin 1. POD (µg/min)	0.0 (0.0; 2.0)	0.00 (0.00; 2.00)	1.00 (0.00; 3.50)	0.468
Milrinone at admission ICU (µg/kg/min)	0.31 (0.21; 0.37)	0.31 (0.21; 0.37)	0.37 (0.29; 0.39)	0.160
Milrinone 1. POD (µg/min)	27.0 (13.2; 27.0)	27.0 (13.2; 27.0)	27.0 (23.4: 27.0)	0.204
Fluid intake <24 h mL)	5530 (3498; 7608)	5360 (3440; 7525)	7830 (5138; 9775)	0.030
AKI KDIGO any stage	44 (41.5%)	36 (36.7%)	8 (100%)	0.001
RRT	31 (30.7%)	23 (24.7%)	8 (100%)	<0.001
Reintubation	21 (18.1%)	19 (17.8%)	2 (22.2%)	0.665
Tracheotomy	44 (37.9%)	36 (33.6%)	8 (88.9%)	0.002
Re-admission to the ICU	11 (9.6%)	10 (9.5%)	1 (11.1%)	1.000
Postoperative delirium	20 (17.1%)	16 (14.8%)	4 (44.4%)	0.045
TIA/Stroke (CT-proofed)	12 (10.3%)	12 (11.2%)	0 (0%)	0.595
CPR	3 (2.6%)	3 (2.8%)	0 (0%)	1.000
Pneumonia	33 (28.2%)	29 (26.9%)	4 (44.4%)	0.268
Sepsis	28 (24.3%)	24 (22.6%)	4 (44.4%)	0.218
Rethoracotomy	33 (28.2%)	29 (26.9%)	4 (44.4%)	0.268
Sternal wound infection/VAC revision	2 (1.7%)	2 (1.9%)	0 (0%)	1.000
Driveline infection	2 (1.7%)	2 (1.9%)	0 (0%)	1.000
ENT bleeding	8 (6.8%)	5 (4.6%)	3 (33.3%)	0.015
GI bleeding	19 (16.2%)	17 (15.7%)	2 (22.2%)	0.638
Cerebral bleeding	6 (5.1%)	6 (5.6%)	0 (0%)	1.000
ICU time (days)	14.5 (6.3; 35.8)	13 (6; 33)	58 (29.5; 71)	0.002
Ventilation time, h	207 (26.5; 946.0)	171 (22; 783)	1206 (810; 1330)	0.001
30 d mortality, %	24 (20.0%)	22 (19.8%)	2 (22.2%)	1.000
Hospital mortality, %	37 (31.9%)	31 (29.0%)	6 (66.7%)	0.029

AKI = acute kidney injury, RRT = renal replacement therapy, ENT = ear-nose-throat, GI = gastrointestinal.

**Table 4:** Comparison of Laboratory Values.

	Total (n = 125)	No SPB (n = 116)	SPB (n = 9)	*p*-Value
Lactate levels prior surgery	1.0 (0.8; 1.3)	1.0 (0.8; 11.4)	0.9 (0.7; 1.3)	0.462
Lactate levels admission ICU	2.9 (1.8; 4.9)	2.9 (1.9; 4.7)	2.9 (1.5; 7.0)	0.995
Lactate levels 1. POD	1.7 (1.2; 2.5)	1.7 (1.2; 2.5)	2.0 (1.2; 4.5)	0.550
Creatinine prior surgery	116.4 (90.0; 171.3)	113.0 (89.6; 158.3)	141.0 (124.0; 190.5)	0.163
Creatinine admission ICU	117.6 (92.1: 167.7)	116.0 (91.6; 164.7)	150.0 (117.5; 181.2)	0.221
Creatinine 1. POD	122.0 (95.0; 168.0)	119.5 (94.7; 168.4)	149.0 (106.0; 176.0)	0.411
CK prior surgery	46.5 (30.8; 114.0)	49.0 (30.5; 118.0)	43.0 (31.0; 145.0)	0.733
CK admission ICU	338 (262; 525)	339.0 (258.5; 531.0)	316.0 (292.5; 464.0)	0.971
CK 1. POD (µmol/L)	463 (272; 819)	464.5 (268.5; 822.3)	421.0 (250.5; 812.5)	0.929
GOT prior surgery	28.9 (19.1; 40.0)	29.1 (18.4; 40.0)	26.9 (21.1; 64.8)	0.733
GOT admission ICU _	59.0 (49.7; 93.1)	60.9 (49.0; 93.6)	57.9 (51.9; 98.9)	0.983
GOT 1. POD	107.0 (67.0; 157.4)	106.9 (66.8; 157.0)	119.0 (96.2; 180.0)	0.439
GPT prior surgery	24.0 (14.7; 48.9)	24.4 (15.0; 49.0)	18.1 (12.8; 48.0)	0.292
GPT admission ICU	24.5 (17.7; 41.5)	24.4 (17.4; 40.5)	24.9 (19.8; 62.7)	0.538
GPT1. POD	28.0 (19.4; 45.1)	27.1 (19.0; 42.0)	38.2 (22.2; 74.5)	0.332
Bilirubin 1 POD	28.8 (16.3; 51.6)	28.8 (15.9; 51.6)	32.4 (21.1; 52.2)	0.359
INR prior surgery	1.30 (1.16; 1.47)	1.30 (1.16; 1.43)	1.41 (1.17; 1.64)	0.349
INR admission ICU	1.18 (1.09; 1.31)	1.18 (1.09; 1.29)	1.23 (1.05; 1.57)	0.568
INR 1. POD	1.18 (1.10; 1.31)	1.18 (1.10; 1.32)	1.20 (1.13; 1.29)	0.933
INR_3. POD	1.27 (1.18; 1.40)	1.26 (1.18; 1.37)	1.55 (1.20; 2.06)	0.055
CRP prior surgery	14.4 (4.2; 47.4)	13.5 (4.2; 46.6)	14.9 (3.3; 58.5)	0.922
CRP admission ICU	76.4 (50.6; 112.2)	75.8 (50.4; 113.7)	93.4 (74.5; 108.0)	0.413
CRP 3. POD	176.5 (111.3; 238.5)	179 (111; 242)	139.0 (110.2; 200.5)	0.383
WBC prior surgery	7.23 (5.75; 9.88)	7.22 (5.77; 9.89)	7.34 (4.80; 10.09)	0.742
WBC admission ICU	14.00 (9.83; 19.03)	14.32 (9.98; 19.05)	13.66 (8.14; 19.64)	0.655
WBC 1. POD	11.45 (7.92; 14.67)	11.63 (7.86; 14.68)	9.67 (7.76; 15.11)	0.659
WBC 3. POD	10.57 (7.79; 14.16)	10.35 (7.78; 14.18)	11.15 (7.96; 14.13)	0.588
Platelet count prior surgery	174.5 (120.5; 219.8)	179.0 (122.0; 228.5)	140.0 (78.5; 213.0)	0.311
Platelet count admission ICU	*153.0 (119.5; 189.5)*	*154.5 (119.3; 192.5)*	*139.0 (117.5; 161.5)*	*0.332*
Platelet count 1. POD	*130.0 (98.0; 160.8)*	*130.0 (99.5; 167.0)*	*119.0 (55.5; 149.0)*	*0.459*
Platelet count 3.POD	*96.0 (74.5; 119.3)*	*96.0 (74.0; 119.5)*	*95.0 (69.5; 120.5)*	*0.904*
Hemoglobin prior surgery	*10.3 (9.4; 11.6)*	*10.3 (9.3; 11.7)*	*9.6 (9.3; 10.3)*	*0.201*
Hemoglobine admission ICU	10.4 (9.3; 11.4)	10.4 (9.4; 11.4)	9.0 (8.7; 10.4)	0.032
Hemoglobin 1. POD	*9.6 (9.1; 10.5)*	*9.6 (9.0; 10.4)*	*10.0 (8.2; 11.5)*	*0.577*
LDH 1. POD	*412.0 (348.5; 528.0)*	*412.0 (339.8; 528.0)*	*442.0 (362.8; 572.3)*	*0.665*
LDH3. POD	*362.0 (300.0; 464.0)*	*362.5 (295.8; 452.0)*	*348.0 (329.0; 585.0)*	*0.392*

**Table 5:** Description of blood and coagulation products administered during SPB.

SPB onset after Implantation (d)	11 (2; 15)
Duration of SPB (d)	11 (7; 33)
Days of mechanical ventilation until onset of SPB (d)	10 (6; 15)
Number of packed red blood cells during SPB	19 (12; 27)
Number of fresh frozen plasma during SPB	13 (10; 20)
Number of platelets during SPB	6 (4; 12)
Fibrinogen during SPB	4 (2; 8)
PCC during SPB (IE)	2400 (1800; 4200)
Faktor XIII during SPB (IE)	6000 (5000; 11,250)
von Willebrand factor during SPB (IE)	19,000 (4000; 31,000)
Days of von Willebrand factor therapy (d)	8 (4; 27)
Novoseven® during SPB (mg)	0 (0; 0)

d = days, PCC = prothrombin complex concentrate.

## Data Availability

The data presented in this study are available on request from the corresponding author. The data are not publicly available due to legal or privacy issues.
